# Role of sirtuins in attenuating plaque vulnerability in atherosclerosis

**DOI:** 10.1007/s11010-023-04714-2

**Published:** 2023-03-23

**Authors:** Prathosh Velpuri, Vikrant Rai, Devendra K. Agrawal

**Affiliations:** Department of Translational Research, Western University of Health Sciences, 309 E. Second Street, Pomona, CA 91766-1854 USA

**Keywords:** Atherosclerosis, Plaque formation, Plaque vulnerability, Sirtuins, Stable plaque, Unstable plaque

## Abstract

Atherosclerosis is characterized by the development of intimal plaque, thrombosis, and stenosis of the vessel lumen causing decreased blood flow and hypoxia precipitating angina. Chronic inflammation in the stable plaque renders it unstable and rupture of unstable plaques results in the formation of emboli leading to hypoxia/ischemia to the organs by occluding the terminal branches and precipitate myocardial infarction and stroke. Such delibitating events could be controlled by the strategies that prevent plaque development or plaque stabilization. Despite the use of statins to stabilize plaques, there is a need for novel targets due to continuously increasing cases of cardiovascular events. Sirtuins (SIRTs), a family of signaling proteins, are involved in sustaining genome integrity, DNA damage response and repair, modulating oxidative stress, aging, inflammation, and energy metabolism. SIRTs play a critical role in modulating inflammation and involves in the development and progression of atherosclerosis. The role of SIRTs in relation to atherosclerosis and plaque vulnerability is scarcely discussed in the literature. Since SIRTs regulate oxidative stress, inflammation, and aging, they may also regulate plaque progression and vulnerability as these molecular mechanisms underlie the pathogenesis of plaque development, progression, and vulnerability. This review critically discusses the role of SIRTs in plaque progression and vulnerability and the possibility of targeting SIRTs to attenuate plaque rupture, focusing on the highlights in genomics, molecular pathways, and cell types involved in the underlying pathophysiology.

## Introduction

Since the beginning of the twentieth century, cardiovascular diseases (CVD) have remained the most common cause of death, taking significant hold, especially in industrialized countries in Western Europe and North America [1]. CVD contributed to 17.5 million deaths annually, approximately 31% of global mortality [2]. Increasing prevalence of CVD conditions such as strokes and ischemic heart disease decreases Disability Life Adjusted Years (DALYs) due to high-blood pressure, diabetes, obesity, poor nutrition, and lack of exercise. This ever-growing concern demonstrates the need for additional therapies in treating CVD [2].

Atherosclerosis is the primary cause and determinant of CVD. It is characterized by luminal occlusion of arteries formed by heavy extracellular fat and lipid deposition and thrombus precipitation onto intimal walls of major arteries and develop into atheromatous plaques [3]. This buildup of plaques narrows the arteries, restricting the volume and flow of blood, and leading to ischemia and hypoxia [4]. Coronary artery disease, myocardial infarction (MI), strokes, and peripheral artery disease are the major manifestations of atherosclerosis when left untreated.

**Fig. 1:**
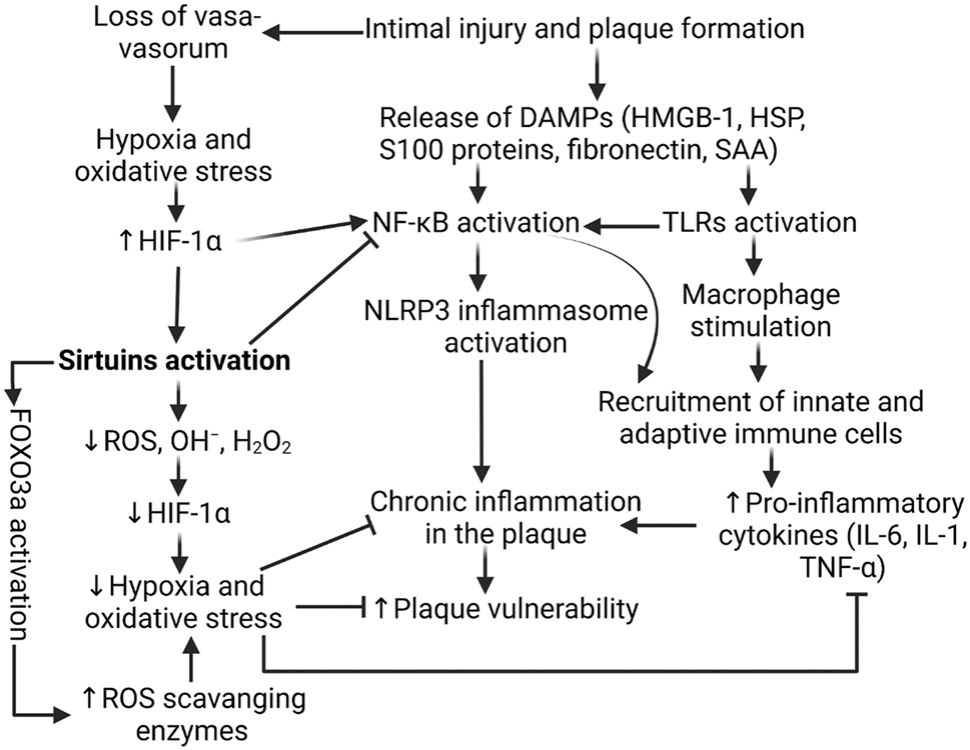
Inflammatory pathway leading to cascade resulting in plaque vulnerability and the effect of Sirtuins attenuating pro-inflammatory mediators**.** Intimal injury during vascular intervention or during atherogenesis induce release of DAMPs which stimulate inflammatory signaling by activating Toll-like receptors (TLRs) and receptor for advanced glycation end-products (RAGE) leading to immune response involving increased recruitment of innate and adaptive immune cells, secretion of pro-inflammatory cytokines, and NLRP3 inflammasome activation. Altogether, they cause chronic inflammation in the plaque and induce vulnerability. Intimal injury also causes disruption of vasa-vasorum and leads to hypoxia and oxidative stress which in turn activate sirtuins. Increased sirtuins act as oxidative stress scavenger and reduce oxygen radicals and decrease oxidative stress which may lead to attenuated plaque vulnerability

Atheromatous plaques in general are of two types: stable (non-vulnerable) and unstable (vulnerable) plaques [5]. The common factors mediating plaque vulnerability are size and depth of injury to the intima, levels of reactive oxidative species (ROS), number of macrophages and lymphocytes present, low-density lipoproteins (LDL) levels, artery size, and blood pressure in the lumen [6]. Among these factors, endothelial cell (EC) dysfunction or injury during vascular intervention is an important risk factor mediating plaque formation. The injury along the intimal layer results in the release of damage-associated molecular patterns (DAMPs) which produce downstream activation of an inflammatory cascade of receptors such as Trigering Receptor Expressed on Myeloid Cells 1 (TREM-1) and Toll-like receptors (TLRs) playing a critical role in atherosclerosis and plaque vulnerability [7]. The activation of these receptors induce increased secretion of inflammatory cytokines such as Tumor Necrosis Factor (TNF)-α, interleukins IL-1 and IL-6, and protease-like matrix metalloproteinases (MMPs), specifically MMP-9 [8] (Fig. 1). The presence of these inflammatory markers makes the plaque more susceptible to damage [9, 10]. Although anti-inflammatory therapies are crucial in reducing atherosclerosis, acute inflammation is vital as it plays a key role in the regeneration of the arterial lining at the time of the earliest lesion. However, when acute inflammation sustains for a long time, chronic inflammation that is maladaptive to the lumen develops and causes the progression of stable plaque to unstable plaque [11, 12]. Along with inflammation, various other factors including hypoxia, oxidative stress, calcification, and neoangiogenesis play a critical role in plaque vulnerability [13]. This review focuses on correlating the possible role of sirtuins in plaque vulnerability.

Additionally, increased expression of HIF-1α leads to activation of NF-κB and thus targeting sirtuins may attenuate oxidative stress and inflammation and enhance plaque stability. High Mobility Group Box 1 (HMGB-1), Heat Shock Protein (HSP), Nuclear factor kappa-light-chain-enhancer of activated B cells (NF-κB), NLR family pyrin domain containing 3 (NLRP3), Interleukin-1 (IL-1), Interleukin-6 (IL-6), Tumor Necrosis Factor-α (TNF-α), Hypoxia Inducible Factor (HIF)-α, Reactive Oxidative Species (ROS), Hydroxyl Radical Ion (OH^−^), Hydrogen Peroxide (H_2_O_2_), Forkhead Box O3 (FOXO3).

## Oxidative stress and sirtuins

Intimal injury, the predisposing factor for plaque formation and progression, and vascular inflammation induce local upregulation of the renin-angiotensin system [14, 15]. Renin–angiotensin–aldosterone system (RAAS) is a highly studied hormonal mechanism that elicits change throughout the body primarily in response to decreased renal perfusion of filtrate and electrolytes. Secretion of renin elicits the release of Angiotensin II (AngII) which serves to increase blood pressure and fluid volume by systematic vasoconstriction [16]. Along with this, Ang II also contributes to several other mechanisms such as in the presence of chronic inflammation, AngII significantly increases free radicals and ROS [17]. Local activation of RAAS also enhances the activation of NF-*κ*B and release of inflammatory cytokines such as IL-6 through AngII, providing a positive feedback loop leading to higher levels of AngII, perpetuating vascular inflammation and plaque vulnerability [18]. Lowering AngII levels may mediate important anti-inflammatory and regenerative vascular tissue growth to stabilize plaque formation.

Oxidative stress is involved in plaque progression, which is partly regulated by the Sirtuin family proteins. Sirtuins have a wide range of roles such as energy metabolism, transcription regulation, DNA repair, circadian rhythm regulation, and more importantly inflammation through their nicotinamide adenine dinucleotide-dependent deacetylase activity (SIRT1-7) and function to suppress gene transcription epigenetically [19]. The role of various sirtuins, their epigenetic mechanism, targets, and biological effects have been reviewed earlier [20]. Among SIRT1 to SIRT7, nuclear sirtuins including SIRT 1, 2, 6, and 7 play a critical role in regulating inflammation; SIRT1 is known to suppress NF-κB, COX-2, and iNOS production, SIRT2 deacetylates p65 subunit of NF-κβ and RIP-1, and SIRT6 interacts with p65/RelA bound to the NF-κβ promoter region and represses transcription [21]. Acute and chronic inflammation alter metabolism, bioenergy reprogramming, and homeostasis which ultimately lead to increased ROS production. The alteration in glycolysis and fatty acid metabolism is linked with NAD + dependent function of sirtuins [22]. SIRT1, whose expression is regulated by various upstream activators and suppressors operating on the transcriptional and post-transcriptional levels, involves in modulating inflammation via its biological effect by deacetylating various proteins and post-translational modifications [23]. In addition to playing a role in homeostasis and inflammation, sirtuins may play role in cellular senescence and aging by regulating insulin/IGF-1 signaling pathway, AMP-activated protein kinase, and forkhead box O (FOXO) [24]. Sirtuins are known to be localized in different regions throughout the cell including cytosol, nucleus, and mitochondria with SIRT1 mainly in nucleus, SIRT2 in cytosol, and SIRT3, 4, and 5 in mitochondria. SIRT3 is also located in the cytosol and nucleus [25]. SIRT3 is closely tied to lipid metabolism and oxidative stress. SIRT3, a stress‐responsive deacetylase, is involved in mitochondrial metabolism and homeostasis, and protects cells from genotoxic and oxidative stress‐mediated cell death [26, 27]. SIRT3 levels change during oxidative stress, genotoxic stress, metabolic stress, and stroke in order to maintain homeostasis to protect the cells [28]. SIRT3 also plays a role in aging and is associated with longevity and it has recently been documented that exercise-induced SIRT3 decreases cellular stress and may contribute to longevity [29]. Sirtuins are also involved in regulating glucose-6-phosphate dehydrogenase (G6PD) activity (SIRT2), SOD2 activity (SIRT3), suppressing inflammation by suppressing AP-1 signaling (SIRT1, 3, and 6), regulating inflammatory signaling in dendritic cells, which play a critical role in plaque vulnerability, thereby regulating T cells and Tregs cells population (SIRT1), inhibiting NLRP3 inflammasome (SIRT1 and SIRT2), and promoting osteoblast differentiation and bone formation (SIRT1, 6, and 7) [10, 30, 31]. With the important role of sirtuins in inflammation, cellular stress, and aging, it is important to delineate the role of sirtuins in plaque vulnerability as inflammation, cellular stress, and aging are the risk factors in plaque vulnerability and atherosclerosis.

Sirtuins expression at the transcriptional level is regulated by transcription factors FOXOs and during nutritional stress SIRT1 expression is regulated by FOXO3a [32]. However, during oxidative stress, sirtuins can regulate the expression of FOXO3a by deacetylation causing increased expression of downstream target genes involved in mitochondrial homeostasis, anti-apoptosis, and anti-oxidative stress [30]. The role of FOXOs in atherosclerosis is also supported by the fact that FOXOs are involved in vessel development, growth, maintenance, and function. FOXOs are also involved in controlling tissue differentiation, growth and maintenance, cell cycle progression, ROS detoxification, programmed cell death, and glucose metabolism [33]. FOXOs also play a role in aging and age-related metabolic disorder like diabetes mellitus type-2 (DMII) and both aging and DMII are risk factors for atherosclerosis [34].

Forkhead box transcription factors (FOXOs), which are regulated by the Sirtuin family and implicated to be an anti-oxidant mediator in many processes, have been implicated in decreasing age-related mortality through the breakdown of ROS and regulation of cell death [35]. Release of ROS from inflammatory mediators is known to increase atheromatous plaque size and pressure by thinning out the fibrotic cap overlying vascular tissue, characterizing vulnerable plaque, suggesting a possible method of attenuating plaque vulnerability by targeting inflammation via the downregulation of oxidative stress imposed by ROS through SIRT3-downstream effect on FOXOs [6] (Fig. 1). However, there is a lack of clear understanding of the underlying molecular mechanisms involving sirtuins and FOXOs and the relationship between these protective measures [36]. Studies on the role of SIRT1 and SIRT2 in histone demethylation and deacetylation in response to immediate stress and cellular death have given reasons to implicate the role of SIRT3 in epigenetic regulation, but it remains controversial. Clear relationships in the expression levels of FOXO3 and SIRT 1–2 and SIRT 6–7 have also similarly implied the role of SIRT3 in specifical regulation of FOX03a. Since epigenetic factors may also affect plaque development, it is important to investigate and understand how sirtuins can affect plaque vulnerability as they have histone modification capability [37, 38]. The following sections are focussed on critically discussing the probable role of sirtuins in regulating plaque vulnerability and the possible therapeutic implications.

## Plaque development and plaque rupture

**Fig. 2:**
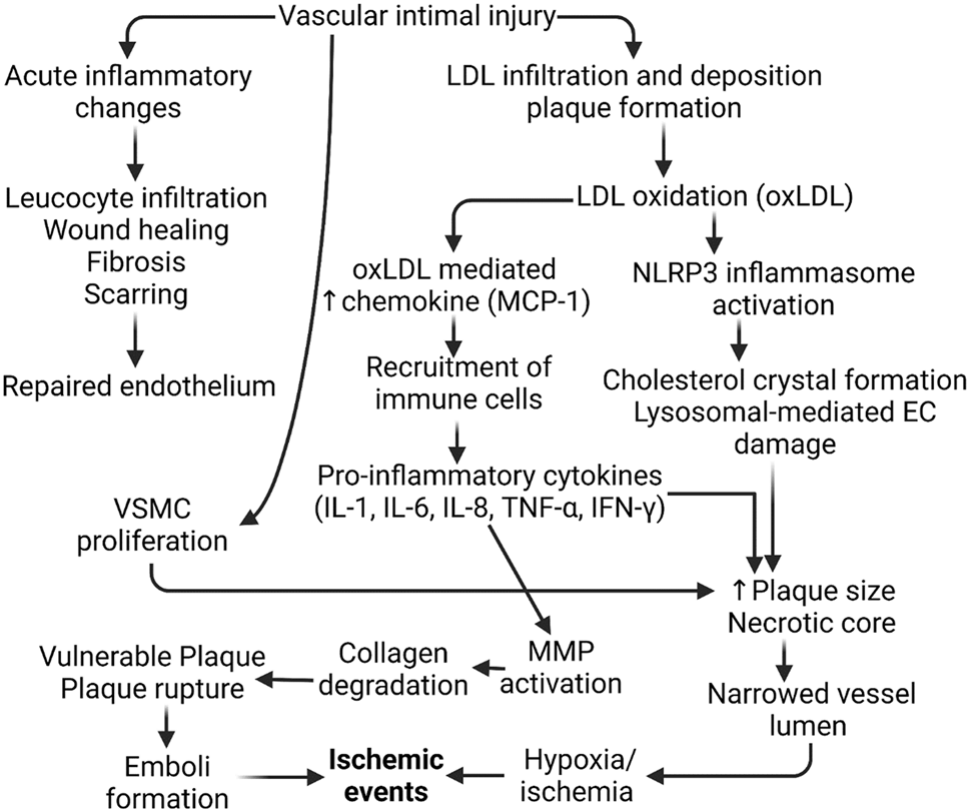
Pathological sequence of Ischemic events in the cardiovascular system resulting from vascular injuries and acute inflammation is gone awry. Oxidized Low-density Lipoprotein (oxLDL), NLR family pyrin domain containing 3 (NLRP3), Interleukin-1 (IL-1), Interleukin-6 (IL-6), Interleukin-8 (IL-8), Tumor Necrosis Factor-α (TNF-α), Interferon-γ (IFNγ)

With the advanced technology capable of classifying atheromatous plaques to be rupture-prone, major addendums have been provided through extensive research on the molecular mechanisms of these plaques. It is thought that micro-injuries to the intimal layer of the artery lead to breaches in the vascular barrier that protect endothelial cells [39]. This breach can lead to deposition and infiltration of various extracellular matrices, collagen, and lipids such as triglycerides and cholesterol carried by various lipoproteins in plasma. LDL accumulation and developing ischemia can result in the recruitment of macrophages by the release of chemoattractants which attempt to prevent further accumulation of cholesterol. As the plaque develops, the intima becomes further leaky, promoting further LDL recruitment, suggesting that high LDL or cholesterol count is a major risk factor for developing atherosclerosis [40] (Fig. 2). Furthermore, several studies showed how epigenetic factors such as both microRNA and long-noncoding RNA help in the regulation of cholesterol efflux, lipid metabolism, and control of inflammation.

About 75% of all coronary events such as strokes and MI stem from plaque rupture and erosion following a period of arterial necrosis and ischemia. These rupture-prone plaques have been classified as vulnerable and unstable [5]. Plaque rupture depends on many internal and external factors of the arterial lining, but generally, it is thought to depend on plaque structural stress (PSS) crossing the threshold of maximal strength [41]. Inflammatory cytokines can promote additional oxidation, necrosis of cellular tissue, and extracellular matrix degradation and attenuate collagen synthesis to break down the integrity of the fibrous cap [42]. This loss of integrity of the fibrous tissue can lead to luminal compromise, which is healed but cause further narrowing [43] (Fig. 2). This suggests that advanced plaques develop in a significantly different manner from early lesions as advanced plaques have higher remodeling rates than earlier plaques. Narrowing of the luminal lining forms regions of high blood-shear stress, mechanically damaging the intimal lining. With this reasoning, stable plaques can be characterized by small or no necrotic cores, intact fibrous caps, and few calcification nodules. Unstable or vulnerable plaques are characterized by thinning of the fibrous cap, presence of necrotic core, chronic inflammation, decreased vascular smooth muscle cells, decreased collagen and other extracelluar matrix, and neo-angiogenesis [8].

Clinical factors like high blood pressure, high cholesterol, diabetes, poor nutrition, and stress are known to drive the development of atherosclerosis by increasing the LDL composition, number, and size as well as arterial permeability to be infiltrated and susceptibility to being inflamed [44]. Since atherosclerosis is a multifocal condition, vulnerable plaques at the risk of rupture and thrombosis in one region can be a marker of advanced cardiovascular disease elsewhere in the body: with regions at the highest risk being coronary arteries [4]. These findings reemphasize the importance of preventing the worsening of stable plaques at the earliest time point to decrease the risk of rupture and thrombosis and resulting coronary events. Since inflammation precedes and superimposes plaque rupture, understanding and targeting the inflammatory pathway to reduce the chance of cardio-ischemic events remain essential [45].

## Loss of protective mechanisms in chronic inflammation and plaque vulnerability

As lipid molecules deposit and are endocytosed into macrophages and dendritic cells, the vascular endothelium necrotizes and forms a fibrotic cap. Tissue hardening and vascular remodeling promote the release of pro-inflammatory molecules, leading to inflammation [46]. Macrophages, after significant uptake of oxidized LDLs and other lipids, become foam cells. Although there are myriad mechanisms of modifications in LDLs and macrophages, nitric oxide (NO) is a key pro-oxidant that is produced by both endothelial cells and macrophages. The effect of NO can be both protective and atherogenic, depending on its source and dosage. However, through inducible NO synthase expressed in foam cells, overproduction of NO lead to dysfunction of the local vasodilatory effects meant to protect the intima from occlusion [47]. Increased macrophage foam cell formation leads to increase oxidative stress and ROS production causing endothelial dysfunction and increased endothelium permeability allowing increased entry of low density lipoproteins (LDL) [48]. Increased LDL deposition enhances plaque vulnerability, thus attenuating oxidative stress by targeting sirtuins may attenuate oxidative stress and thereby reduced LDL deposition and plaque vulnerability [49]. This notion is supported by the fact that SIRT2 significantly decreases plaque area, macrophage infiltration, expression of iNOS and increases the levels of ARG-1 (arginase-1) in the atheroma of LDLR knockout mice and enhance plaque stability by attenuating macrophage polarization towards M1 phenotype [50].

In periods of non-infectious inflammation, loss of intima prompts the release of DAMPs such as high-mobility group box 1 (HMGB1), heat-shock proteins (HSPs), fibronectin, and serum amyloid A (SAA). These DAMPs induce inflammation by activating downstream signaling involving the NF-κB pathway or directly stimulating macrophages by binding to Toll-like receptors (TLRs), particularly TLR4 [51]. Chemoattractants released by macrophages at the site also recruit other components of the innate and adaptive immune systems [52] (Fig. 1). Initially, macrophages were thought to be the primary key players in plaque development. However, later studies revealed the combined effect of CD4 + T cells, regulatory T cells, and myeloid cells that produce immature macrophages and mast cells. In normal vascular regenerative processes, mast cells and CD4 + T cells are not present in significant numbers, as the concentration of cytokines to recruit these cells is not sufficient. Mast cells in vulnerable plaques overexpress vascular endothelial growth factor (VEGF), leading to the overdevelopment of collagen and ECM layer, promoting the hardening of the necrotic tissue. Continued overexpression of TLR4 and its downstream signaling cascades create an abnormal positive feedback loop of intimal lesion and inflammation, rendering the plaque to be further infiltrated and more vulnerable [53]. Therefore, the breakdown of the protective effects of acute inflammation, presence of persistent inflammation, inflammatory immune cell recruitment, and functional disorders in developing atherosclerotic plaque mediate the loss of protective mechanisms and plaque vulnerability [8]. In the case of neuroinflammation in Alzheimer’s (AD), triggering DAMPs activate a cascade of NF-κB and its subsequent downstream inflammatory pathways and the formation of NLRP3 inflammasomes, both of which are critically involved in inflammation related to atherosclerosis [54] (Fig. 1). Several studies in the neuroinflammation of AD showed that Sirtuins, specifically SIRT1 and SIRT2, exhibit protective effects by targeting DAMPs and downregulating NF-κB, yielding sirtuins as a possible therapeutic target [55]. DAMPs and other pattern recognition receptors (PRR) can further propagate hypoxia-mediated disorders as described in studies investigating TLR4 signaling in obstructive sleep apnea [56]. Hypoxia is cited to be one of the important mediators in plaque vulnerability as lower oxygen potentials caused by stenotic arteries lead to higher expression of IL-1β and caspase-1 activation, along with altering lipid metabolism [57]. Chronicity of inflammation due to dysregulated innate and adaptive immune response plays a critical role in plaque progression, thrombosis, and vessel stenosis [58].

Expression and activation of NF-κB is one of the central events of inflammation, and once stimulated by TREM-1, TLR4, and DAMPs, NF-κB translocates to the nucleus to regulate transcriptional activity to increase pro-inflammatory molecules. One such molecule that is activated by NF-κB is IL-1β, which proteolytically activates a multiportion assembly called inflammasome, in particular the NLRP3 inflammasome [54]. Inflammasomes, in general, are responsible for sensing cellular stressors such as cellular damage, electrolyte imbalance, and ROS levels. Components of NLRP3 inflammasomes are highly expressed in foam cells within atheromatous plaques, additionally enhanced through low oxygen tension and aggravating inflammation (Fig. 2). Along with indirectly increasing NLRP3 levels through IL-1β, NF-κB can proteolytically activate NLRP3 in foam cells [59]. In addition to regulating other proteins, NF-κB can also be modulated by protein modifications such as phosphorylation, methylation, and acetylation; one such epigenetic regulator is the Sirtuin family.

## Hypoxia and plaque vulnerability

ROS such as superoxide (O^2−^) and hydroxyl (OH·) radicals is a highly reactive molecule class that is derived from aerobic metabolism. Homeostasis of ROS is a key determinant factor of many organisms and affects their capacity to produce energy from coupling mechanisms [60]. As cells regularly age, apoptosis and proper cleanup of ROS are handled in a very complex, but regulated manner. However, at the sites of necrosis and chronic inflammation, ROS can be released in the ECM, forming foam cells, and increasing the size of the fibrotic cap (Fig. 2). One such enzyme that has been a key player in the overproduction of ROS is NADPH oxidase, which also induces autophagy. Autophagy involves cells rapidly degrading old cellular components to generate a nutrient pool to remodel as well as remove damaged mitochondrial organelles. Autophagy has also been involved with lipid homeostasis in blood, further complicating LDL levels in the body as well as activating vascular disorders such as atherosclerosis [61]. It has been shown that defective autophagy significantly stimulates inflammatory responses with the activation of TLR-9 through the release of mitochondrial DNA [62]. Overly active autophagy in response to NADPH oxidase in pre-existing chronic inflammation can induce fibrous membrane formation, detrimentally reducing the nutrient pool, especially in collagen, leading to more vulnerable plaques [63]. Homeostasis of ROS can be key to controlling plaque size.

Occlusion of intimal layers limits blood flow and exchange between blood in the vessels and cells, inducing hypoxia. Although the exact mechanisms of how hypoxia induces and is inducible by ROS are still debated, it seems like Complexes I, II, and III of the electron transport chain leads to the production of ROS which then stabilizes the production of Hypoxia-inducible Factor alpha (HIF1α) [64] (Fig. 2). HIF1α is a major transcription factor involved in tissue regeneration, cellular adaption to low O_2_, as well as regulating NF-κB and Sirtuins. Chronic periods of inflammation will result in prolonged periods of hypoxia throughout the body, spreading inflammation at various vascular sites. NF-κB is dependent on ROS and is expressed to transcript pro-inflammatory genes such as monocyte chemotactic protein (MCP)-1) and IL-6. In vascular smooth muscle cells, Ang II induces MCP-1 through NF-κB. In a similar manner to DAMPs, AngII-induced NF-κB DNA binding and activation causes apoptosis in the media of the blood vessels and mediates an increase in adhesion with MCP-1 [65]. Growth of adhesion molecules, infiltration of inflammatory cells and the development of necrotic tissue due to cell death in this manner directly lead to plaque destabilization and further inflammation, showing that high levels of ROS and AngII can directly play a key role in the development of atherosclerotic plaques (Fig. 2).

It has been found that SIRT1 increases during hypoxia. An increase in HIF1α is shown to increase gene expression of SIRT1 as well as a reduction of SIRT1 levels in HIF1α knockout mice with high sensitivity has been documented [66] (Fig. 1). However, the role of SIRT1 on HIF1α levels is still unknown. Since AngII and ROS largely destabilize plaques through the downstream NF-κB pathway, understanding and investigating the epigenetic regulation of NF-κB through the ROS-SIRT1-FOXO3 axis may be beneficial, and targeting sirtuins and FOXO proteins can attenuate or slow thrombotic plaque pathogenesis [33].

## Molecular mechanisms of FOXOs and sirtuins in plaque vulnerability

Although Sirtuins have various roles in several locations, in general, they produce tissue-protective effects. Numerous cancer studies have shown significant sirtuin involvement in gene silencing and detoxification. For example, SIRT1-6 increase genomic stability by repairing single and double-stranded DNA breaks from oxidative stress from tumor growth [67]. The most studied in the group, SIRT 1 in particular has been implicated with reducing inflammation in multiple cell types undergoing obesity-induced insulin resistance, which involves defects in insulin signaling, systemic inflammation, mitochondrial disruption, and cellular stress [68]. SIRT1 is also cleaved in inflammatory conditions and low NAD + levels during oxidative stress, which then impair SIRT1 signaling [69]. This suggests both the protective and antagonistic role of SIRT1 during inflammatory signaling. One such mechanism in how SIRT1 achieves this is by deacetylating and thus inhibiting the expression of NF-κB [70]. Since inflammation, oxidative stress, and mitochondrial dysfunction play a critical role in the pathogenesis of plaque formation, progression, and atherosclerosis, sirtuins may play a role in plaque vulnerability, however, this remains obscure [71]. What remains unclear about the role of SIRT1 in atherosclerosis is during the periods of hypoxia. There is no yet clear relationship between SIRT1 expression and HIF-1α levels, exhibiting increasing effects in some tissues and decreasing in others [15, 72]. However, it has been demonstrated that SIRT1 inhibits NFκB by deacetylating the p65 subunit of the NF-κB complex as well as activating AMPK, PPARα, and PGC-1α, which then inhibit NF-κB signaling and suppress inflammation [70]. By reducing NF-κB levels, SIRT1 could stabilize vulnerable plaques before excessive intimal lining is inflamed and there is a foam cell overgrowth, making the plaque rupture-prone [73] (Fig. 1).

Although SIRT3 is less widely studied and understood than its counterparts in the Sirtuin family, its location of expression and effects in the mitochondria provide promising hopes for new therapy and alternatives in the field. SIRT3 has similar roles to SIRT1 in being negatively associated with ROS and protecting cells from oxidant-induced cell death [74]. Studies about metabolism and caloric reduction highlight SIRT3 as an essential player in the mitochondrial glutathione antioxidant defense system. A plethora of work that has been done around sirtuins revolves around cancer research, and it has been found that SIRT3 knockout cells have, in fact, higher levels superoxide radicals and are prone to being genetically vulnerable and more likely to develop tumors [75]. More directly, mtDNA induced by higher superoxide levels can predict higher-risk plaques [76].

SIRT3 has also been further identified as a tumor suppressor by decreasing levels of ROS, minimizing HIF1α [77]. There is still a lack of information on whether this relationship extends to arterial narrowing-induced hypoxia. However, by reducing HIF1α levels, SIRT3 could yield protective mechanisms in response to oxidative stress posed by atheromatous plaques as HIF1α levels are directly correlated to plaque pathogenesis [78].

Through cancer research, it has been found that SIRT3 reduces ROS through the activation of transcription factors, particularly FOXO3a in adipocytes by expressing ROS-scavenging enzymes [79]. Significant decreases in ROS following overexpression of SIRT levels have been found in multiple cell types such as age-related auditory issues, cardiomyocytes that have been oxidatively damaged due to doxorubicin, and most particularly endothelial cells undergoing hypoxic stress [80–82]. It has been implied that SIRT3 stabilizes FOXO3a by deacetylation which enhances the mitochondrial antioxidant defense system, and FOX03a levels are reduced in periods of SIRT3 deficiency through HIF1α playing an intermediary role [77].

## Targeting sirtuins to attenuate plaque vulnerability

It has been shown that myeloid deletion of SIRT3 accelerates adipocyte inflammation in cells overexpressing AngII [83]. This could be due to SIRT3 mediating deacetylation of pyruvate dehydrogenase E1 alpha (PDHA1). PDHA1 deficiency correlates with mitochondrial dysfunction and promotes glycolysis, which further creates radical species [84]. AngII levels probably aggravate and accelerate infiltration of foam cells and secretion of inflammatory interleukins in SIRT3 knockout mice, due to the loss of protective mechanisms set in place by the stabilization of FOX03a. As previously discussed, AngII elicits chronic inflammation, exacerbating plaque vulnerability and leading to the growth of necrotic tissue at the vascular lesion. Vulnerable plaques have a high likelihood to rupture, and in most worsened conditions can lead to ischemia-induced events such as MI and strokes. Targeting and utilizing sirtuins, in particular SIRT3, remains a potential therapeutic target to reduce such occurrences. Many such cancer studies have shown potential targets for therapy using Sirtuin Activators (STAC) to target the anti-inflammatory cascade potentiated by sirtuins.

There is growing evidence that cellular aging or cellular senescence can promote atherosclerotic plaques [85]. Cells undergoing senescence cause pro-inflammatory secretion which can lead to loss of tissue-repair capacity. Senescent endothelial cells have an increased likelihood of recruiting monocytes and releasing more tissue factors, leading to coagulation and thrombotic plaques. Originally discovered in aging studies, pharmacological modifiers of sirtuins such as STACs can inhibit senescent cell-mediated secretion of chemokines, particularly by overexpression of SIRT1 [85]. SIRT1 inhibits p53/p21 signaling along with maintaining telomere health, both of which are considered anti-senescent effects [86].

**Fig. 3:**
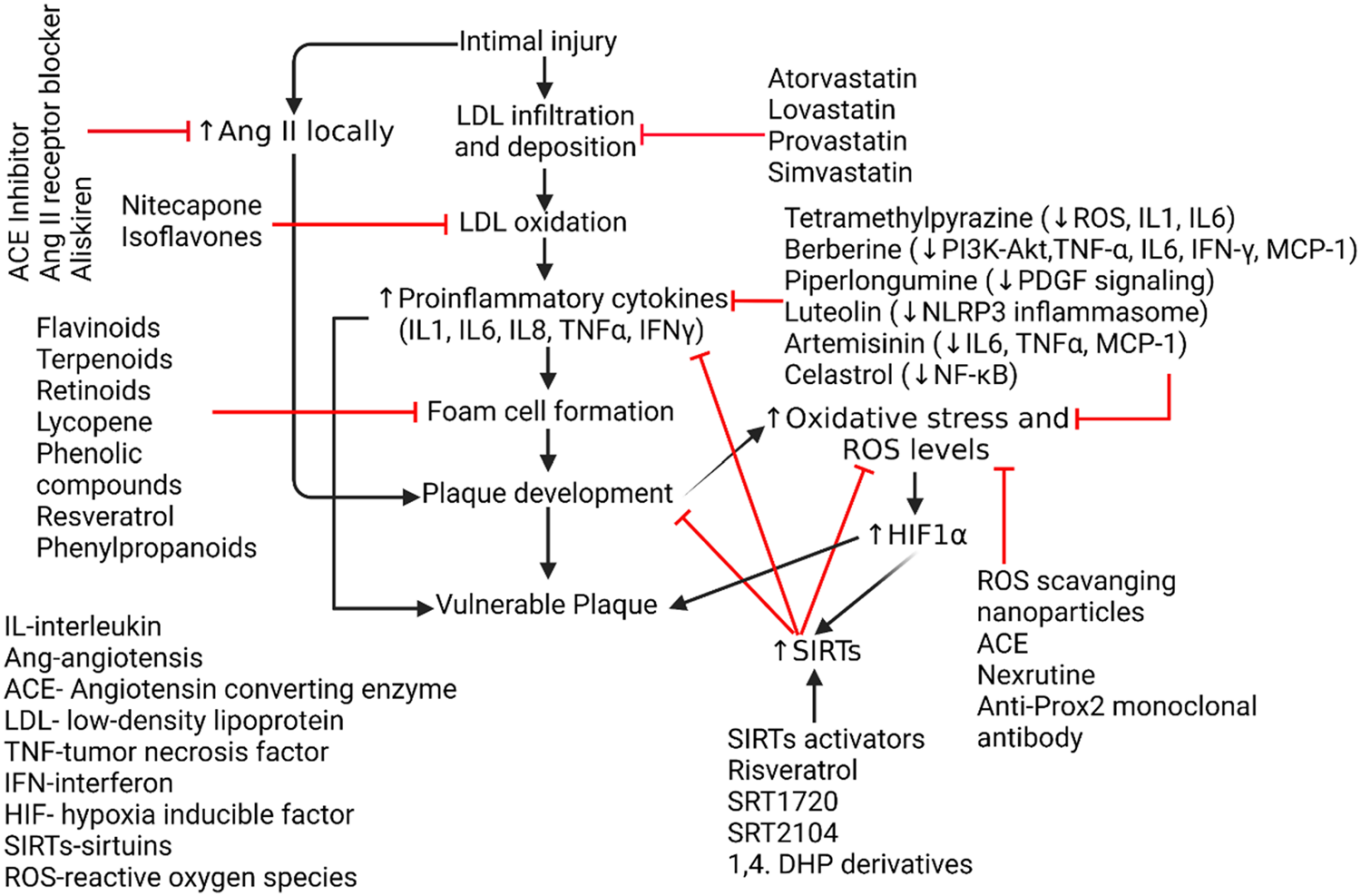
Pharmacological modulators to be used in sequential steps of development of atheromatous plaques. Angiotensin II (AngII), Oxidized Low-density Lipoprotein (oxLDL), NLR family pyrin domain containing 3 (NLRP3), Platelet-derived growth factor (PDGF), phosphoinositide-3-kinase–protein kinase B/Akt (PI3K-PKB/Akt), Monocyte chemoattractant protein-1 (MCP-1/CCL2), SRT (SIRT activator), 1,4 Dihydropyridine (DHP)

The concurrent prevalence of both diabetes and CVD points to insulin regulation as an important mediator of atherosclerotic plaques [87]. In addition to promoting LDL dysregulation, there is increased medial and intimal calcification, which can lead to loss of fibrotic cap. In a study measuring SIRT6 expression in diabetic patients, SIRT6 is very much involved with the inflammatory pathways as it is expressed significantly higher in non-diabetics [88]. This study highlights the loss of sirtuin-led downregulation of calcification, inflammation, and infiltration in diabetes, which leads to a higher likelihood of plaques. Furthermore, SIRT6 overexpression decreased the proliferation of atherogenic genes such as tumor necrosis factor superfamily member 4 (TNFSF4), which may impair the adhesion of monocytes to endothelial cells, directly impairing the formation of the plaques [89]. Targeting SIRTs to attenuate plaque vulnerability and progression of atherosclerosis is also supported by the fact that SIRTs attenuate inflammation by inhibiting NF-κB (SIRT1 and SIRT6), decrease apoptosis by inhibiting p53 by deacetylation (SIRT2), decrease oxidative stress (SIRT3), and regulate LDL by inhibiting PCSK9 (SIRT2) [90]. The results of the studies discussed above and in Table 1 suggest that SIRTs might be important players and therapeutic target in attenuating plaque vulnerability and atherosclerosis progression (Fig. 3) [91].

**Table 1:** Summary of the studies investigating the role of sirtuins (SIRTs) in atherosclerosis

Aim of the study	Experimental model	Strategy	Study outcome
To evaluate the role of SIRT6 in decreasing atheroprogression	ApoE^−/−^ mice fed a high-fat diet	High fat-fed mice were evaluated for plaque and atherosclerosis in SIRT6 KO mice	SIRT6 decreased the TNFSF4 gene by deacetylating H3K9 [89] SIRT6 attenuated monocyte adhesion to ECs
To evaluate and measure SIRT1 levels in CAD and explain risk factors	Human adults	A case–control study with peripheral venous blood samples	SIRT1 is negatively correlated to cholesterol content and risk of CVD [94]
To investigate the role of SIRT6 in smooth muscle cells	SIRT6 Knockout Mice	SMC-specific SIRT6-deficient (SIRT6KO) mice were infused with Ang II to induce oxidative stress	SIRT6KO mice developed aortitis, aortic hemorrhage, and aneurysms in response to Ang II [95]
To investigate the role of SIRT6 in atherosclerotic lesion development	ApoE^−/−^ mice	SIRT6 was knocked down in ApoE^(−/−)^ mice using small hairpin RNAs (shRNAs) lentivirus injection and then evaluated for plaque size and vulnerability	Intracellular adhesion molecule-1 expression was significantly upregulated in aortic endothelium without SIRT6 expression leading to higher monocyte adhesion [96]
To measure the risk of coronary plaques in asymptomatic patients using SIRT1 levels	Human adults	A case–control study between high-risk and non-high-risk groups considering age, total cholesterol, gender	SIRT1 may play a predictive role in plaque screening before coronary angiography [97]
Role of mi-RNA and other epigenetic modulators in atherosclerosis	Human adults	A case–control study between blood samples obtained from healthy patients and individuals with atherosclerosis	SIRT1 is a target of miR-217-mediated downregulation of macrophage apoptosis and subsequent inflammatory response
Role of SIRT6 expression in diabetic patients	Human adults	A case–control study between diabetics and nondiabetic patients undergoing carotid endarterectomy	Higher presence of SIRT1 and downregulation of NF-kB in non-diabetics [88]
Investigate genetic polymorphisms associated with Sirtuins with CAD	Human adults	A case–control study between blood samples obtained from healthy patients and individuals with CAD using genomic DNA	Genetic polymorphisms in SIRT3 and SIRT6 were found in higher correlation with patients with CAD [98]
Role of SIRT3 in vascular inflammation	ApoE^−/−^ mice	Evaluated if endothelial-selective SIRT3 deletion accelerates vascular inflammation and oxidative stress and the protective effect of NAD + to alleviate these changes in endothelial cells using mice model	Upregulation of vascular inflammatory markers and oxidative stress with plaque embolization was found significantly with SIRT3 knockout [99]
Role of SIRT7 in mitochondria stability	*SIRT7*^−/−^ mice	Generation of *SIRT7*^*−/−*^ mice gene targeting strategy in 129 Sv embryonic stem cells	SIRT7 is a dynamic nuclear regulator of mitochondrial function through its impact on GABPβ1 function [100]
		In vitro acetylation and deacetylation assays using GABPβ1 protein	
Role of SIRT3 in cardiac hypertrophy and interstitial fibrosis	SIRT3-deficient and SIRT3-overexpressing transgenic mice	SIRT3-deficient and SIRT3-expressing transgenic mice were exposed to hypertrophic stimuli	SIRT3 suppresses levels of ROS, thus endogenously negatively regulating cardiac hypertrophy [101]
		Cardiomyocytes were used to study the role of FOXO3a in cardiac protection and oxidative stress	
Role of SIRT1 in cellular aging	ApoE^−/−^ mice	Downregulation of SIRT1 in VSMCs isolated from human plaques	SIRT1 regulates DNA damage repair and survival in vascular smooth muscle cells [102]
		SM22-SIRT1^+/+^, SIRT1^+/Δex4^, and SM22-SIRT1^Δex4/Δex4^ littermate mice and ApoE^−/−^ were fed normal chow or high-fat diet	
Role of SIRT2 in pathological cardiac hypertrophy	LDLr^−/−^ mice	Female LDLr^−/−^ mice were treated with saline, empty lentivirus, lentivirus-SIRT2, or shRNA-SIRT2 for 4 weeks	By inhibiting macrophage polarization, SIRT2 inhibited atherosclerotic plaque progression, but the degree of effect is small and not significant [50]
		Atherosclerotic plaques were assessed in the aortic sinus and macrophage polarization was evaluated	
SIRT6 role in Atherosclerosis	Human VSMC and ApoE^−/−^ mice	SIRT6 expression was measured in human VSMC derived from plaques as well as ApoE^−/−^ high-fat diet	SIRT6 expression was reduced in human and mouse plaques and was shown to maintain telomeres while inhibiting atherogenesis [103]

Abbreviations: Ang, Angiotensin; CAD, coronary artery disease; CVD, cardiovascular disease; ECs, endothelial cells; KO, knockout; LDLr, low density lipoprotein receptor; TNFSF4, TNF Superfamily Member 4

The epidemic of COVID-19 has also led to increased number of cases of incident venous thromboembolism, heart failure, and overall cardiovascular deaths [92]. It is suspected that SARS-CoV-2 has potential to accelerate the progression of atheromatous plaques by destabilizing old plaques and inducing further endothelial damage [93]. Host response to the virus includes innate inflammation in the lungs that is largely mediated by alveolar macrophages and can cause onset of acute respiratory distress. Inflammatory responses remain high and consistent for longer times than expected. Interaction between SARS-CoV-2 and ACE2 receptors in endothelium is well noted to reduce levels of ACE2 preventing the degradation of atherosclerotic AngII. The role of AngII in atherosclerosis has been been discussed above, and the effect of Sirtuins in downregulating AngII and anti-oxidant activity can promote the downregulation of pro-inflammatory cytokines to being transported to atheromatous plaques as seen highly in COVID-19 patients.

## Conclusion

Regulation of vascular function is dependent on the balance between the adaptive inflammatory mechanisms in the body that are meant to produce fibrosis, wound healing, scarring, and maladaptive infiltration by pro-inflammatory cytokines. Factors such as diabetes, high cholesterol, high blood pressure, and other adverse cardiac and systemic manifestations can predispose individuals to maladaptive mechanisms. Infiltration by oxidized LDL which can recruit macrophage-derived foam cells, lymphocytes, cholesterol crystal development, and ECM fibrosis can subsequently lead to the generation of an atheromatous plaque. The development and vulnerability of a plaque to rupture are dependent on a combination of the oxidative stress, metabolism, and susceptibility to develop chronic inflammation. Studies have revealed and highlighted targets throughout this inflammatory pathway that can attenuate plaque vulnerability. Outside traditional therapeutic and pharmacological ways of targeting atherosclerosis, emerging studies show that targeting sirtuins through sirtuin activators and modulators can potentially yield significant attenuation of plaque formation and vulnerability. Sirtuins are expressed in highly inflammatory, hypoxic, and cell-cycle dysregulated states, all of which are prone to developing more atheromatous plaques. Regulated epigenetically, sirtuins can be targeted to regulate downstream inflammatory cascade and antioxidant scavengers. In view of the limited literature on the role of sirtuins in regulating vascular health, further investigations are warranted.

## Data Availability

Not applicable since the information is gathered from published articles.
